# A Novel Osteotomy Preparation Technique to Preserve Implant Site Viability and Enhance Osteogenesis

**DOI:** 10.3390/jcm8020170

**Published:** 2019-02-01

**Authors:** Chih-Hao Chen, Benjamin R. Coyac, Masaki Arioka, Brian Leahy, U. Serdar Tulu, Maziar Aghvami, Stefan Holst, Waldemar Hoffmann, Antony Quarry, Oded Bahat, Benjamin Salmon, John B. Brunski, Jill A. Helms

**Affiliations:** 1 Division of Plastic and Reconstructive Surgery, Department of Surgery, Stanford University School of Medicine, Stanford, CA 94305, USA; chchen5027@gmail.com (C.-H.C.); benjamin_coyac@hotmail.fr (B.R.C.); amasaki@stanford.edu (M.A.); bleahy94@gmail.com (B.L.); serdartulu@gmail.com (U.S.T.); maziara@stanford.edu (M.A.); brunsj6@stanford.edu (J.B.B.); 2 Craniofacial Research Center, Department of Plastic and Reconstructive Surgery, Chang Gung Memorial Hospital, Chang Gung University School of Medicine, Taoyuan 33305, Taiwan; 3 Department of Clinical Pharmacology, Faculty of Medical Sciences, Kyushu University, Fukuoka 812-8582, Japan; 4 Nobel Biocare Services AG P.O. Box, CH-8058 Zürich-Flughafen, Switzerland; stefan.holst@nobelbiocare.com (S.H.); waldemar.hoffmann@nobelbiocare.com (W.H.); antony.quarry@nobelbiocare.com (A.Q.); 5 Department of Prosthodontics, School of Dentistry, Johann-Wolfgang Goethe University, 60438 Frankfurt, Germany; 6 Private Practice, Beverly Hills, CA 90210, USA; odedbahat@gmail.com; 7 Paris Descartes-Sorbonne Paris Cité University, EA2496 Montrouge, France; benjamin.salmon@parisdescartes.fr; 8 Dental Medicine Department, Bretonneau Hospital, HUPNVS, AP-HP, 75018 Paris, France

**Keywords:** osteogenesis, osteotomy, bone healing, bone chips, drilling tool design

## Abstract

The preservation of bone viability at an osteotomy site is a critical variable for subsequent implant osseointegration. Recent biomechanical studies evaluating the consequences of site preparation led us to rethink the design of bone-cutting drills, especially those intended for implant site preparation. We present here a novel drill design that is designed to efficiently cut bone at a very low rotational velocity, obviating the need for irrigation as a coolant. The low-speed cutting produces little heat and, consequently, osteocyte viability is maintained. The lack of irrigation, coupled with the unique design of the cutting flutes, channels into the osteotomy autologous bone chips and osseous coagulum that have inherent osteogenic potential. Collectively, these features result in robust, new bone formation at rates significantly faster than those observed with conventional drilling protocols. These preclinical data have practical implications for the clinical preparation of osteotomies and alveolar bone reconstructive surgeries.

## 1. Introduction

The medical and dental professions, with few exceptions, adapted commercially available tools for use that were developed for drilling other materials [1]. For example, bone-cutting tools, which are largely predicated on the design of metal-cutting instruments. Metal drills are end-cutting tools, e.g., only the tip of the drill is engaged in producing a hole, and the same is true for the vast majority of bone-cutting drills [2]. Metal drills and most bone drills are also designed to cut at a high rotational velocity, which means that the drill can be advanced with minimal axial thrust force [3]. Metal and bone drills generally have a relatively small rake angle, which means that particles generated by cutting are typically scattered from the site to avoid obstructing the drill. Metal drilling typically requires a lubricant that serves as a coolant [4]; in bone cutting, these functions are replaced by saline irrigation [5]. 

We studied the biological responses to osteotomy site preparation in multiple animal species [6,7,8,9] including humans [10], and these analyses, coupled with computational and finite element modeling [5], prompted us to reconsider the design of a bone-cutting tool, optimized for osteotomy site preparation. The resulting tool, called the OsseoShaper, is designed to limit osteocyte death caused by mechanical and thermal damage, and simultaneously retain osseous coagulum/bone chips generated by bone cutting. For this study, cutting tools were downscaled to accommodate the smaller size of the rat maxillae; however, the ratio of cutting-tool diameter and bone surface area was representative of what is used clinically. The purpose of this study was then to compare osteotomies produced by a downscaled OsseoShaper versus a conventional drill in terms of heat generation, osteocyte viability, bone remodeling, and onset of new bone formation. 

## 2. Materials and Methods

### 2.1. Animals and Experimental Plan

Stanford APLAC approved all procedures (#13146), which conform to ARRIVE guidelines. In total, 18 female Wistar rats (Charles River Laboratories) were used in this study. All animals underwent ovariectomy (OVX) and bilateral maxillary first molar (M1) extraction when they were seven weeks old. Animals were then maintained for eight weeks, during which time the osteoporotic phenotype developed [11,12] and the extraction socket completed its healing [9]. All animals were then subjected to bilateral osteotomy site preparation in the healed M1 location. Animals were sacrificed at intervals of 0.5 days, three days, and seven days. Before surgery, general anesthesia was reached via intraperitoneal injection of ketamine (100 mg/kg) (Vedco, Inc., St. Joseph, MO, USA) and xylazine (10 mg/kg) (Akorn, Inc., Lake Forest, IL, USA), while analgesia was reached via subcutaneous injection of Buprenorphine SR (0.5 mg/kg). After surgery, rats recovered in a controlled, 37 °C environment, fed a soft food diet for the duration of the experiment and housed in groups of two. Weight changes were <10%. No adverse events (e.g., uncontrolled pain, infection, prolonged inflammation) were encountered.

### 2.2. Ovariectomy and Tooth Extraction

To align our experimental model with the average patient receiving a dental implant, e.g., >50 years old [13], seven-week-old female rats underwent OVX [14]. This produced in our animal model an osteopenic/osteoporotic phenotype, which is representative of patients over 50 years of age [15]. In brief, a dorsal midline incision was made between the mid-back and tail base. The peritoneal cavity was accessed through bilateral muscle layer incisions, the ovary was identified, and the connection between the fallopian tube and the uterine horn was suture-ligated. After bilateral removal of the ovaries, the wounds were closed layer by layer. 

In parallel with the OVX, bilateral maxillary first molars (M1) were extracted. This further aligned our experimental model with patients, in which the majority of dental implants are placed in healed extraction sites [16]. In brief, micro-forceps were used to loosen and remove the tooth in toto. Bleeding was controlled by local compression. Healing of the extraction site was confirmed using histology and micro-computed tomographic (µCT) imaging. By post-extraction day 21 (PED21), the extraction site was fully healed, as shown by the fact that the bone volume/total volume (BV/TV) of the extraction site was equivalent to adjacent, pristine bone [10].

### 2.3. Osteotomy Site Preparation 

To directly compare two surgical drilling tools for their ability to maintain osteotomy site viability, rats were anesthetized before a full thickness periosteal flap was elevated at the M1 tooth extraction site. A handpiece (KaVo Dental, Uxbridge, UK) with saline irrigation was used to produce a pilot 1.0-mm drill hole, followed by step-wise enlargement using progressively larger drill diameters (Table 1). In the OsseoShaper protocol, the same type of pilot drill was used to produce a pilot osteotomy; thereafter, a downscaled prototype of the OsseoShaper was used to enlarge the osteotomy to the same final maximum diameter as was achieved with the conventional surgical drill protocol. The mini OsseoShaper was used without irrigation. Drill speeds were adjusted to result in the same radial velocity for all drills and to compensate for slightly different diameters. Each osteotomy was made with a new drill. After osteotomy, tension-free primary closure of the periosteal flap was achieved using tissue glue (VetClose, Henry Schein, Dublin, OH, USA).

### 2.4. Tissue Collection and Processing

Tissues were collected at post-osteotomy day (POD) 0.5 to evaluate micro-damage and programmed cell death caused by surgical drilling, as well as at POD3 and POD7, when new bone formation is initiated [7]. In brief, animals were euthanized; then, the entire maxillae were dissected free from other tissues and transferred to 4% paraformaldehyde (PFA) and stored at 4 °C overnight. After fixation, samples were decalcified in ethylenediaminetetraacetic acid (EDTA), embedded in paraffin, and sectioned at an 8-μm thickness for analyses. Tissue sections were deparaffinized in Citrisolv (Decon Labs, Inc., King of Prussia, PA, USA) and hydrated via a series of decreasing concentrations of ethanol before staining or other histological/cellular activity analyses.

### 2.5. Histology 

For aniline blue staining, sections were treated with a saturated solution of picric acid followed by a 5% phosphotungstic acid solution and staining in 1% aniline blue. Slides were then dehydrated and mounted using Permount (Fisher Scientific, Hampton, NH, USA). For pentachrome staining, sections were pre-treated with 6% nitric acid and stained with toluidine blue solution for 5 min (0.5 g toluidine blue in 100 mL of distilled water at pH 1 to 1.5, adjusted with 0.5% HCl). Picrosirius red staining [17] was used to detect collagenous osteoid matrix. Tissues were stained with picrosirius red then viewed under polarized light. Tightly aligned fibrillary collagen molecules appear red compared to less organized collagen fibrils that show a color of shorter (green–yellow) wavelengths.

### 2.6. Quantification of Programmed Cell Death

Terminal deoxynucleotidyl transferase deoxyuridine triphosphate (dUTP) nick end labeling (TUNEL) staining (Roche Diagnostics GmbH, Mannheim, Germany) was performed according to the manufacturers’ guidelines. Following deparaffinization and rehydration, paraffin sections were stained by incubating slides in permeabilization solution for 8 min, adding TUNEL reaction mixture, then incubating at 37 °C in the dark. Between these steps, paraffin sections were washed with phosphate-buffered saline (PBS). To quantify the extent of apoptotic osteocytes, TUNEL-stained tissue sections from 4–6 different samples were analyzed. Each section was photographed using a Leica digital image system at 20× magnification. The number of TUNEL-labeled osteocytes corresponding to apoptotic cells was determined, and the cells grouped according to their distance from the cut edge. The corresponding area for each group was then calculated. The number of apoptotic cells per unit area was calculated by dividing the number of apoptotic cells to the corresponding area (cell/mm^2^).

### 2.7. Tartrate-Resistant Acid Phosphatase (TRAP) Activity 

Identification of osteoclasts was done using TRAP staining. TRAP activity was observed using a leukocyte acid phosphatase staining kit (catalog #386A-1KT, Sigma-Aldrich, St. Louis, MO, USA). Tissue sections were processed according to the manufacturer’s instructions. To quantify the TRAP activity, TRAP-stained tissue sections were photographed using a Leica digital image system at 10× magnification. The TRAP^+ve^ area corresponding to osteoclasts was determined within the radial zone extending 300 µm from the cutting edge. The TRAP^+ve^ ratio was calculated by dividing the TRAP^+ve^ pixels by the total pixels of the region of interest.

### 2.8. Immunostaining 

To localize, within the osteotomies, cells that had initiated differentiation down an osteogenic lineage, immunostaining was performed using standard procedures [18]. In brief, following deparaffinization, endogenous peroxidase activity was quenched by 3% hydrogen peroxide for 5 min, and then washed in PBS. Slides were blocked with 5% goat serum (Vector S-1000) for 1 h at room temperature. The appropriate primary antibody was added and incubated overnight at 4 °C, then washed in PBS. The primary antibodies used in this study were Osterix (1:1200; ab22552, Abcam, Cambridge, MA, USA) and Cathepsin K (1:200; ab19027, Abcam, Cambridge, MA, USA). Samples were incubated with appropriate biotinylated secondary antibodies (Vector BA-x) for 30 min, then washed in PBS. An avidin/biotinylated enzyme complex (Kit ABC Peroxidase Standard Vectastain PK-4000, Vectorlabs, Burlingame, CA, USA) was added and incubated for 30 min, and a 3,3′-diaminobenzidine (DAB) substrate kit (Kit Vector Peroxidase substrate DAB SK-4100, Vectorlabs, Burlingame, CA, USA) was used to develop the color reaction. Phalloidin immunostaining was performed using Palloidin Control, DyLight 488 conjugate (1:300; PI21833, Invitrogen, Grand Island, NY, USA). 

### 2.9. Histomorphometric Analyses 

Histomorphometric measurements were performed using ImageJ software v.1.51 (NIH, Bethesda, MD, USA). To quantify the amount of new bone formation in the osteotomy site as a function of time, a minimum of four osteotomy sites were analyzed. For each osteotomy site, a minimum of six aniline blue-stained histologic sections that spanned the distance from the furcation to the apex were used to quantify new bone formation. Each section was photographed using a Leica digital image system at 20× magnification. To calculate the percentage of new bone formation, the number of aniline blue^+ve^ pixels within an osteotomy was measured and divided by the number of the total pixels in the same osteotomy area.

### 2.10. Micro-Computed Tomography (µCT)

Scanning and analyses followed published guidelines [19]. Three-dimensional µCT imaging was performed at various times after surgery. In brief, samples were fixed in 4% PFA at 4 °C overnight. Then, they were transferred to 70% ethanol solution for µCT scanning before the decalcification process. A µCT tomography data-acquisition system (VivaCT 40, Scanco, Brüttisellen, Switzerland) at 10.5-μm voxel size (70 kV, 115 μA, 300 ms of integration time) was used for scanning and reconstruction. Bone morphometry was performed using the acquisition system’s analysis software (Scanco). Multiplanar reconstruction and volume rendering were carried out using Avizo (FEI, Hillsboro, OR, USA) and ImageJ v1.51 (NIH, Bethesda, MD, USA) software, before being imported into Adobe Photoshop and Illustrator (CC2017, Adobe, San Jose, CA, USA). 

### 2.11. Calculation of Osteotomy Surface Roughness

To calculate the irregularity of the osteotomy walls, the Shape Filter plugin for ImageJ was employed [20]. Ten transverse sections were used to outline the contours of osteotomies using ImageJ. The contours were then converted to black-and-white images, and the plugin was used to obtain the convexity and solidity values. Convexity measured the surface roughness of a two-dimensional (2D) shape and was defined as H/P, where H was the perimeter of convex hull of the shape, and P was the perimeter of the contour. Solidity was defined as C/A, where C was the area occupied by the contour, and A was the area occupied by the convex hull of the contour. The perimeter of the contour was defined as the total length of the shape’s perimeter. Shapes such as a square have equal lengths of convex hulls and perimeter of the contours, which results in a convexity = 1. A star, however, has a pentagon convex hull (consider the shape when surrounded by a rubber band) while the perimeter of the contour is the star shape itself. Since the perimeter of the star contour (P) is larger than the convex hull (H), its convexity (H/P) is <1 and, therefore, its surface is rougher compared to a same-sized pentagon. 

### 2.12. Heat Transfer During Drilling

The temperature produced when cutting with a conventional protocol involving multiple drills was compared to site preparation with the mini OsseoShaper. Sawbones 35 (Pacific Research Laboratory, Vashon Island, DC, USA) was used. Drills and drilling protocols used are as listed in Table 1. Thermal radiation was measured immediately after drilling via an infrared camera (SEEK CompactPRO, Seek Thermal Application, Santa Barbara, CA, USA). The drilling protocol was repeated six times in new Sawbones. Means and standard deviations were reported.

The temperature distribution during drilling was also calculated in MATLAB using a finite difference method. Details of the heat transfer model are described in Reference [5]. The differences between the conventional drill and mini OsseoShaper models can be summarized as follows: in the conventional high-speed drilling, the heat flux was applied to the drill hole’s boundary where the tip of the drill was located, and the tip was moved vertically. Below the drill tip, the value of the heat flux was set to zero, and irrigation was applied above the drill tip. In the OsseoShaper low-speed drilling, the heat flux was applied to the drill hole’s boundary at and above the tip due to the tapered shape of the drill, such that the points of engagement between the drill and the bone increased over time as the drill was moved vertically.

### 2.13. Statistical Analyses 

Results were presented in the form of means ± standard deviations, with *N* equal to the number of samples analyzed. Student’s *t*-tests were performed. Significance was set at *p* < 0.05, and all statistical analyses were performed with SPSS software (IBM, Armonk, NY, USA).

## 3. Results

### 3.1. A New Surgical Drilling Tool That Cuts Efficiently at Very Low Speeds 

**Figure 1:**
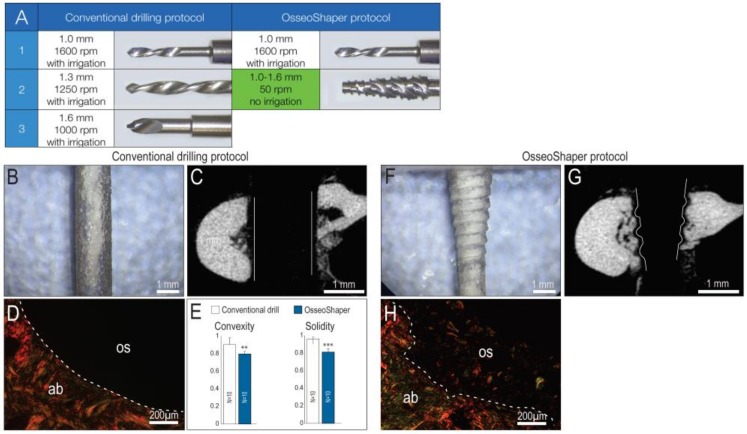
Osteotomy site preparation with OsseoShaper requires fewer steps and, unlike conventional drills, produces a rough surface. (**A**) All osteotomy site preparations began with the use of a 1.0-mm pilot drill run at 1600 rpm plus irrigation; afterward, the conventional osteotomy procedure used a 1.3-mm drill (1250 rpm plus irrigation) followed by a 1.6-mm drill (1000 rpm plus irrigation). The OsseoShaper protocol used the same 1.0-mm pilot drill at 1600 rpm plus irrigation, and was then followed by the OsseoShaper run at 50 rpm without irrigation. Using a conventional drill (**B**) in plexiglass demonstrates the shape and texture of a cut surfaces, and (**C**) in bone, µCT sections illustrate the parallel walls of the osteotomy. (**D**) Picrosirius red staining of a representative transverse tissue section demonstrates the resulting smooth cut surface. (**E**) Quantification of surface texture, as expressed by convexity and solidity, resulting from a conventional drilling protocol. Using an OsseoShaper (**F**) in plexiglass demonstrates a tapered shape with a threaded surface, (**G**) which is validated by µCT imaging. (**H**) Picrosirius red staining of a representative transverse tissue section demonstrates the textured cut surface and the retention of collagen containing osseous coagulum. Solid and dotted lines show the edge of the osteotomy. Two asterisks indicate *p* < 0.01. Three asterisks indicate *p* < 0.001. Scale bars (**B**,**C**,**F**,**G**) = 1 mm, and (**D**,**H**) = 200 μm. Abbreviations: ab, alveolar bone; os, osteotomy.

Most osteotomies are produced through the stepwise enlargement of an initial pilot drill hole with sequentially larger diameter drills [21], all coupled with the use of copious irrigation [22]. We recapitulated that clinical scenario in a rat model, by producing osteotomies using surgical drills with progressively larger diameters. The final drill was 1.6 mm in diameter and was run at 1000 rpm with irrigation (Figure 1A). In osteotomies produced with the downscaled prototype of OsseoShaper, the same pilot drill hole was produced and then followed by a single drill, the OsseoShaper (Figure 1A). The OsseoShaper was run at 50 rpm without irrigation. 

A conventional surgical drill is designed to cut only at its tip, which produces a smooth-walled osteotomy, visible both in plexiglass (Figure 1B) and µCT section of bone (Figure 1C). Analyses using picrosirius red staining revealed, under polarized light, the collagen organization at the cut edge when a conventional drill was employed (Figure 1D). Quantification of surface texture, as expressed by convexity and solidity, resulting from a conventional drilling protocol demonstrated the smoother cut edge (Figure 1E). By contrast, the OsseoShaper was designed with a cutting flute running its length; this resulted in a heteromorphic, textured osteotomy surface, visible both in plexiglass (Figure 1F) and in µCT (Figure 1G). Picrosirius red staining demonstrates the textured cut surface and the retention of collagen containing osseous coagulum (Figure 1H).

### 3.2. The OsseoShaper Allows the Retention of Viable, Autologous Bone Chips in the Osteotomy

**Figure 2:**
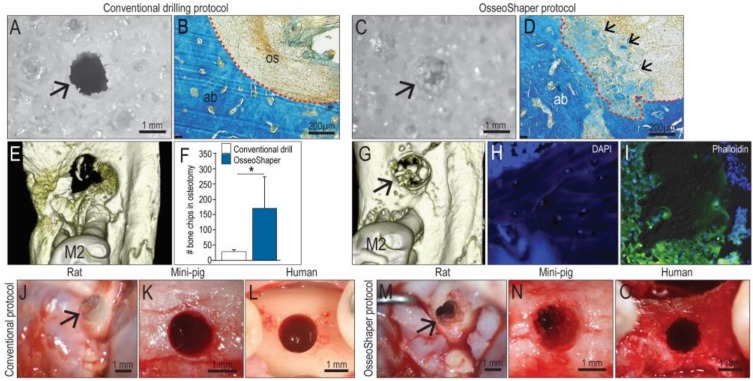
OsseoShaper-produced osteotomies retain more viable bone chips and osseous coagulum. Gross view of a hole produced in 0.32 g/cc Sawbone prepared with (**A**) a conventional drilling protocol versus (**C**) OsseoShaper. Representative transverse sections stained with aniline blue in the osteotomy sites using (**B**) a conventional drilling protocol and (**D**) OsseoShaper protocol. Micro-CT imaging of an osteotomy prepared with (**E**) a conventional drilling protocol versus (**G**) an OsseoShaper. (**F**) Quantification of bone chips in the osteotomy by µCT imaging (*N* = 5). Representative tissue sections of bone chips produced by the OsseoShaper using (**H**) 4′,6-diamidino-2-phenylindole (DAPI) and (**I**) phalloidin staining. Intra-operative view of an osteotomy prepared with conventional drills versus the OsseoShaper in rats (**J**,**M**), in mini-pigs (**K**,**N**), and in patients (**L**,**O**). Arrows indicate the osteotomy. Small arrows in (**D**) indicate the osteoid matrix. Dotted lines show the edge of the osteotomy. Asterisk indicates *p* < 0.05. Scale bars = 1 mm (**A**,**C**,**J**–**O**) and 200 μm (**B**,**E**). Abbreviations: as indicated previously.

Conventional drills have a rake angle that ranges from 0 to approximately 5°, the consequence of which is the production of small (<30 µm) bone particles. In addition, conventional drills are typically run at rotational velocities of 800 rpm or higher [23]. Finally, conventional drills are designed to rotate in the same direction, regardless of whether they are being advanced or withdrawn from the osteotomy. Collectively, these attributes result in minimal retention of particulate matter, as can be visualized when cutting Sawbone in vitro (Figure 2A). Coupled with irrigation, the majority of bone debris is typically removed from the osteotomy (Figure 2B). 

By comparison, the rake angle on a mini OsseoShaper produces osseous coagulum and relatively large (~100 µm) bone chips; additionally, the OsseoShaper is designed to be reversed upon removal. These features result in the collection of bone chips in the cutting flutes, which are then transferred into the osteotomy while the tool is being withdrawn. These events can be visualized when cutting Sawbone (Figure 2C), and upon histologic examination of the osteotomy using aniline blue staining to detect osteoid matrix (Figure 2D). 

Micro-CT imaging was used to quantify the volume of osseous coagulum and bone chips retained in the osteotomy. These analyses verified that OsseoShaper osteotomies retained significantly more osseous material than did conventionally prepared osteotomies (Figure 2E,F,G). A closer examination of the bone chips produced by the OsseoShaper using 4′,6-diamidino-2-phenylindole (DAPI; to detect viable cells) and phalloidin staining (to detect actin filaments) revealed that a subset of chips retained viable osteocytes within the osseous matrix (Figure 2H) and that the majority of chips were surrounded by viable cells (Figure 2I). Clinically, bone chips were only visible in the OsseoShaper-prepared osteotomy sites; this aspect was consistent among species, including rats, mini-pigs, and humans (Figure 2J–O).

### 3.3. The Mini OsseoShaper Preserves Peri-Implant Bone by Limiting Heat Transfer and Minimizing Thermal Apoptosis 

**Figure 3:**
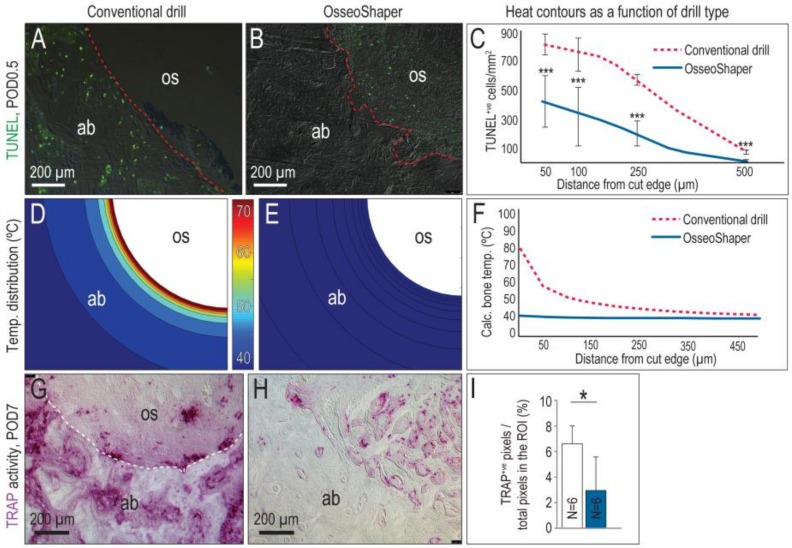
The OsseoShaper generates less heat, which results in a smaller zone of cell death and less tartrate-resistant acid phosphatase (TRAP)-mediated bone remodeling than conventional drills. (**A**) Representative tissue section from an osteotomy prepared using standard drills, where terminal deoxynucleotidyl transferase deoxyuridine triphosphate (dUTP) nick end labeling (TUNEL)^+ve^ cells are apoptotic osteocytes. (**B**) Equivalent tissue section from an osteotomy prepared using the OsseoShaper, where the majority of apoptotic cells are detected in the osseous coagulum. (**C**) Distribution of TUNEL^+ve^ cells as a function of distance from cut edge of osteotomy. Computational models were used to map the distribution of heat in bone as a function of distance from the cut edge, in osteotomies produced using (**D**) conventional drills and (**E**) using the OsseoShaper. (**F**) Calculated temperatures in bone, expressed as a function of radial distance from conventional drills (dotted red line) and from the OsseoShaper (blue line) (*N* = 6). (**G**) Representative transverse tissue section from an osteotomy produced with conventional drills, analyzed on for TRAP activity on post-osteotomy day 7 (POD7). (**H**) TRAP activity on a representative transverse tissue section from an OsseoShaper-produced osteotomy. (**I**) Quantification of TRAP^+ve^ pixels/total pixels in the region of interest (ROI). A dotted line is used to indicate the cut edge of the osteotomy. Asterisk indicates *p* < 0.05. Scale bars = 200 μm. Abbreviations: as indicated previously.

A zone of osteocyte death is produced by conventional drilling [6,7,10]. For example, cutting at 1000 rpm with irrigation produced a ~250-µm-wide, circumferential distribution of TUNEL^+ve^, apoptotic osteocytes (Figure 3A, quantified in C). By comparison, minimal osteocyte apoptosis was detected after OsseoShaper site preparation (Figure 3B, quantified in C). 

A computational model was used to calculate peak temperatures produced by both types of cutting tools, taking into account the speed at which the drills were run, the density of the bone being cut, and, in the case of conventional drill protocols, the use of irrigation [5]. In the case of conventional protocols, a peak temperature of ~80 °C was generated at the cut edge, despite the use of copious irrigation (Figure 3D; quantified in F). Temperatures decreased as a function of distance from the cut edge but nevertheless, temperatures were >40 °C within a ~150-µm circumferential zone (Figure 3A and [6]). 

By comparison, the mini OsseoShaper generated significantly lower (~40 °C) peak temperatures (Figure 3E; quantified in F). Even without the use of irrigation, calculated temperatures immediately adjacent to the cut edge remained in the physiologic range (Figure 3F), well below temperatures known to cause osteocytes necrosis, i.e., 45 °C [24]. 

An in vitro method supported our conclusion that drilling with the mini OsseoShaper produced less heat. Using Sawbones, site preparation was carried out following the same protocol as used for the site preparation in the rat maxilla (Figure 1A) and, immediately thereafter, the temperature of each drill was measured using an infrared camera (Figure S1, Supplementary Materials). The same method was used to measure heat radiating from the mini OsseoShaper. In the conventional protocol, the heat radiating from conventional drills was significantly higher for each step compared to the heat radiating from the mini OsseoShaper (Figure S1).

Osteocyte death is typically accompanied by peri-implant bone resorption [6,7]. In the case of osteotomies produced with conventional drills, the osteoclast marker TRAP was detected throughout the bone adjacent to the osteotomy edge, as well as in the osteotomy itself (Figure 3G). By contrast, mini OsseoShaper osteotomies exhibited minimal TRAP-mediated bone resorption (Figure 3H). The TRAP activity that was detected reflected new bone remodeling in the osteotomy (Figure 3H; quantified in I).

### 3.4. In Mini OsseoShaper Osteotomies, New Bone Formation Is Accelerated 

**Figure 4:**
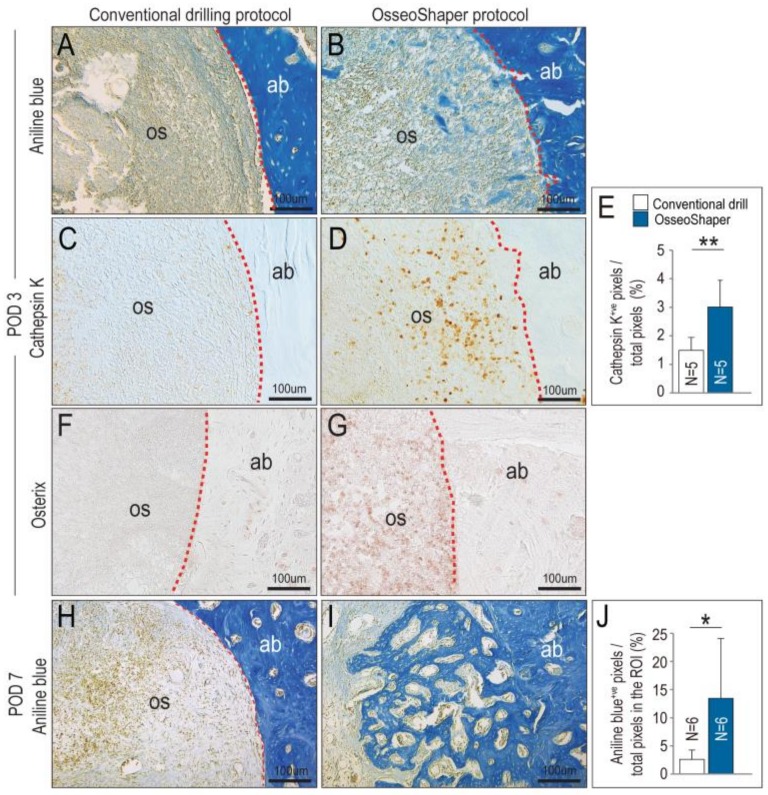
OsseoShaper drilling protocol promotes alveolar bone healing. Representative transverse tissue sections stained with aniline blue on post-osteotomy day 3 (POD3) following osteotomy site preparation with (**A**) conventional drills versus (**B**) the Nobel OsseoShaper. Note the presence of osseous coagulum in the osteotomy site prepared with the OsseoShaper. Adjacent tissue sections immunostained with Cathepsin K in the osteotomy sites of (**C**) conventional drills versus (**D**) the Nobel OsseoShaper. (**E**) Quantification of Cathepsin K^+ve^ pixels/total pixels in the osteotomy site. Adjacent tissue sections immunostained with Osterix in the osteotomy sites of (**F**) conventional drills versus (**G**) the OsseoShaper. (**H**) Tissue sections stained with aniline blue show minimal new bone formation in conventional drill group, while (**I**) osteotomies in the OsseoShaper group show more new bone formation on POD7. (**J**) Quantification of aniline blue^+ve^ pixels/total pixels in the osteotomy site. Dotted lines show the edge of the osteotomy. One asterisk indicates *p* < 0.05. Two asterisks indicate *p* < 0.01. Scale bars = 100 μm. Abbreviations: as indicated previously.

The OsseoShaper was designed to retain osseous coagulum, e.g., mineralized particles including cortical and trabecular bone chips, blood, and stroma that have inherent osteogenic potential [25,26]. On POD3, evidence of this retained osseous coagulum was abundant; compared to conventionally prepared osteotomies, those prepared with the mini OsseoShaper were filled with aniline blue^+ve^ osteoid matrix (compare Figure 4A,B). This matrix served as a nidus for new bone formation and remodeling, as demonstrated by significantly higher Cathepsin K (Figure 4C,D; quantified in E) and Osterix (Figure 4F,G) expression in the mini OsseoShaper osteotomies. By POD7, mini OsseoShaper osteotomies were filled with new bone at a time point when conventionally prepared osteotomies had not yet started to repair (Figure 4H,I; quantified in J).

## 4. Discussion

Most reconstructive surgeries involve the cutting and removal of bone tissue [27] and, ideally, the goal is to resect a well-defined volume of bone and leave behind a cut edge that is favorable to early cell attachment and matrix deposition [28,29]. Clinicians universally agree that the preservation of cell viability is of utmost importance [30,31,32], and that high-speed rotating instruments can compromise this viability because they create thermal and mechanical trauma [33,34,35,36,37]. Irrigation can reduce some of the heat generated by high-speed rotatory surgical drills [22,38], but irrigation also removes bone chips, connective tissue stroma, blood, and stem-cell populations, collectively referred to as osseous coagulum, which have osteogenic potential [39,40,41,42].

The importance of preserving bone viability led to the development of a wide variety of new cutting tools for bone [43]. For example, gas and solid-state lasers use linear thermal absorption to ablate osteoid tissues and, while they are effective at removing the bone, they also generate heat and consequently show many of the same detrimental effects as drilling [44,45]. Plasma ablation lasers avoid some of these problems by creating energy pulses in very small (i.e., µm) zones that result in very high (several thousand Kelvin) temperatures over a very short (picosecond) duration. The result is limited thermal damage to the bone [46]; technical constraints, however, limit the use of these lasers in most clinical practices [47]. 

The OsseoShaper was designed to efficiently cut bone at a low (<50 rpm) velocity. This low-speed drilling results in less bone being cut per unit time and, therefore, less heat evolution per unit time (Figure 3). Less heat generation by the OsseoShaper translates into less of a temperature rise in the bone, which obviates the need for a coolant (Figure 3). The biological sequelae of lower heat generation by the mini OsseoShaper was shown by analyses for osteocyte apoptosis and osteoclast activity; because of the minimal temperature rise, few osteocytes underwent programmed cell death, which translated into less peri-implant bone resorption (Figure 3). Clinicians are fully aware that a viable osteotomy site is critical for new bone formation, and this point is perhaps best illustrated by the extent to which surgeons will go to reduce heat produced by rotary cutting tools. Here, we show that improved osteotomy site viability is indeed directly related to enhanced osteogenesis, which we believe will logically translate into a faster osseointegration of an implant placed into such osteotomies.

### 4.1. A Unique Design That Enables Retention of Bone Chips and Osseous Coagulum in an Osteotomy Site 

Most drills produce bone chips and osseous coagulum, which has inherent osteogenic material that can stimulate new bone formation [25,26]. Most of this osteogenic material is flushed out of the site by irrigation [25], which is required to cool conventional drills. The rake angle of the OsseoShaper produces larger bone chips than conventional drilling protocols. 

Most conventional drills rotate clockwise, whether advancing or withdrawing the tool and, coupled with the high rotational velocity, effectively disperse the bone chips and osseous coagulum. The OsseoShaper slowly rotates clockwise when advanced and is then reversed upon withdrawal; this design feature effectively retains bone chips and osseous coagulum in the osteotomy site (Figure 2). This feature was also visible in osteotomy site preparation performed in mini-pig and human individuals (Figure 2). Historic studies demonstrated that such bone chips that remain in situ are highly osteogenic [48]. 

Cutting flute placement affects the roughness of the osteotomy. Compared to the smooth-walled osteotomies produced by conventional drills, osteotomies produced by the OsseoShaper are textured (Figure 1). Some investigators speculated that a textured surface represents an optimal site for new bone deposition because it mimics the bone surface left behind after osteoclast-mediated remodeling [49]. 

Clinicians recognize that a bone graft from a patient has osteogenic potential and, therefore, a variety of methods were developed in an attempt to collect this autologous material [50]. Most, if not all, of these collection methods necessitate removal of the autologous bone chips from the body and storage ex vivo. In doing so, the bone graft material is potentially subjected to desiccation, temperature changes, e.g., deviations from 37 °C, and bacterial contamination. Use of the OsseoShaper negates these concerns; bone chips remain in situ and, in doing so, their viability is likely to be enhanced and/or preserved.

### 4.2. A Streamlined Protocol for Osteotomy Site Preparation

In conventional drilling protocols, a pilot hole is first produced; then, the osteotomy is gradually enlarged through the use of progressively larger diameter drills. A pilot hole is also created before use of the OsseoShaper, after which the final sized osteotomy is produced in a single step (Figure 1). In conventional drilling protocols, the use of multiple drills increases the chance of deviating from the intended axis of the osteotomy, which in turn impacts the axis of the implant placed into the osteotomy [51]. By reducing the number of surgical drills required to produce the final osteotomy, the alignment error is also effectively reduced [52], and subsequent implant placement will follow the axis of the last drill. 

## 5. Conclusions

In our study, we present a new drill design that is meant to efficiently cut bone at a very low rotational speed, obviating the need for irrigation as a coolant and a lubricant. Osteocyte viability is maintained by the low-speed cutting that produces little heat. Autologous bone chips are generated and maintained on site thanks to the lack of irrigation, coupled with the unique design of the cutting flutes. This osseous coagulum has inherent osteogenic capacities. Collectively, a robust formation of new bone is observed with the new drill design, at rates significantly faster than those observed with conventional drilling protocols. These data have practical applications for clinical implant site preparation and alveolar bone reconstruction.

## Figures and Tables

**Table 1:** Surgical drill parameters.

Company	External Diameter	Product Identifier
OsteoMed	1.0 mm	220-0065
OsteoMed	1.3 mm	220-0064
OsteoMed	1.6 mm	220-0116
Downscaled prototype of the OsseoShaper	1.0 mm (apex) 1.6 mm (crest)	Non-commercial prototype

## Supplementary Materials

The following are available online at http://www.mdpi.com/2077-0383/8/2/170/s1, Figure S1: Thermal radiation measurements of conventional and Osseoshaper protocols in Sawbones.
