# Supplementary material for: A Novel Osteotomy Preparation Technique to Preserve Implant Site Viability and Enhance Osteogenesis
Source: J Clin Med. 2019 Feb 1;8(2):170. doi: 10.3390/jcm8020170 (PMC6406409; doi:10.3390/jcm8020170)
Supplement: Supplementary file 1 [file jcm-08-00170-s001.pdf]

## Supplemental data

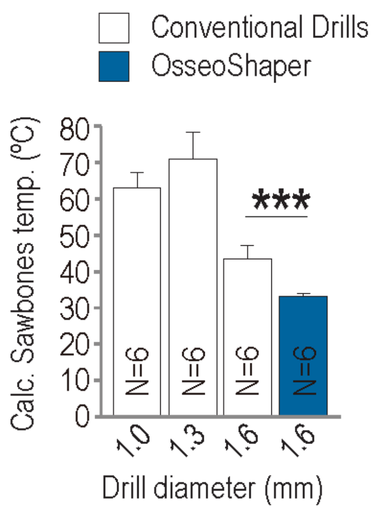


**Figure S1.** Thermal radiation measurements of conventional and Osseoshaper protocols in Sawbones. In the conventional protocol, the heat radiating from conventional drills was significantly higher for each step compared to the heat radiating from the mini OsseoShaper. Three asterisks indicate $p<0.001$.
